# Genome Editing Technologies as Cellular Defense Against Viral Pathogens

**DOI:** 10.3389/fcell.2021.716344

**Published:** 2021-07-15

**Authors:** Yingzi Zhang, Mo Li

**Affiliations:** Biological and Environmental Sciences and Engineering Division (BESE), King Abdullah University of Science and Technology (KAUST), Thuwal, Saudi Arabia

**Keywords:** genome editing, viral infectious disease, CRISPR/Cas, SARS-CoV-2, HIV, hepatitis B, emerging pathogen

## Abstract

Viral infectious diseases are significant threats to the welfare of world populations. Besides the widespread acute viral infections (e.g., dengue fever) and chronic infections [e.g., those by the human immunodeficiency virus (HIV) and hepatitis B virus (HBV)], emerging viruses, such as severe acute respiratory syndrome coronavirus 2 (SARS-CoV-2), pose great challenges to the world. Genome editing technologies, including clustered regularly interspaced short palindromic repeats (CRISPR)-CRISPR-associated (Cas) proteins, zinc-finger nucleases (ZFNs), and transcription activator-like effector nucleases (TALENs), have played essential roles in the study of new treatment for viral infectious diseases in cell lines, animal models, and clinical trials. Genome editing tools have been used to eliminate latent infections and provide resistance to new infections. Increasing evidence has shown that genome editing-based antiviral strategy is simple to design and can be quickly adapted to combat infections by a wide spectrum of viral pathogens, including the emerging coronaviruses. Here we review the development and applications of genome editing technologies for preventing or eliminating infections caused by HIV, HBV, HPV, HSV, and SARS-CoV-2, and discuss how the latest advances could enlighten further development of genome editing into a novel therapy for viral infectious diseases.

## Introduction

**FIGURE 1:**
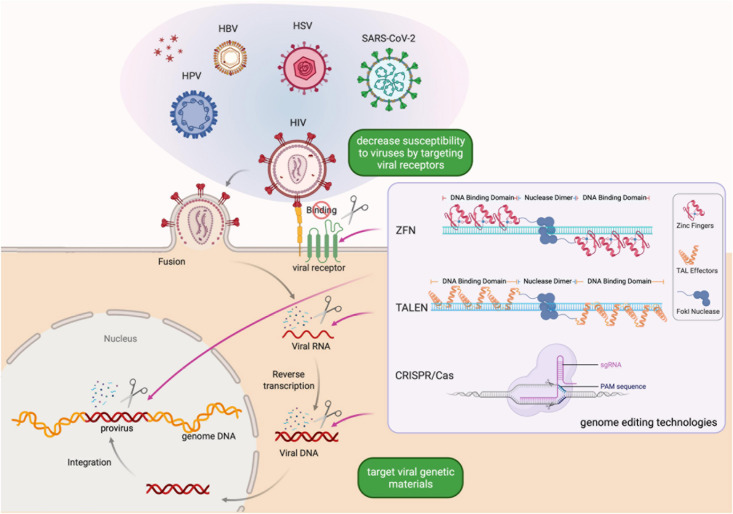
Schematic of genome editing strategies for preventing or eliminating viral infections. Genome editing-based antiviral strategy is simple to design and can be quickly adapted to combat infection by a wide-spectrum of viral pathogens, aiming to eliminate latent infections and provide resistance to new infections. Created with BioRender.com.

Viral infectious diseases are significant threats to human well-being and a global economic burden (King et al., 2006). Though the life cycle of different viruses may vary, it typically starts with the attachment of viruses to target cells and is followed by cellular entry, uncoating to release viral contents, replication and biosynthesis guided by the viral genome, assembly of new viral particles, and release of virions. By completing its life cycle, the virus multiplies and can infect more cells. Besides, many viral pathogens can establish latent or chronic infections by integrating their genome into the genome of the host cell (Figure 1). Since the integrated viral genome, called provirus, remains dormant in the host cell and its daughter cells, it can evade humoral immunity or antiviral treatment and become reactivated and assemble new virions under favorable conditions. Thus, despite of the development of antiviral therapy, chronic infection is still uncurable for many viral pathogens (Trickey et al., 2017; Kulkarni et al., 2020; Pujanandez, 2020). Effective treatment options are currently absent for many viral infectious diseases.

The emergence of genome editing technologies enables researchers to precisely manipulate specific genomic sequences. By adding, removing, or altering specific DNA sequences in the genome, genome editing technologies offer new solutions for the treatment of viral infectious diseases in the clinic. The mainstream genome editing technologies include zinc finger nucleases (ZFNs), transcriptional activator-like effector nucleases (TALENs), and clustered regularly interspersed short palindromic repeats and CRISPR-associated proteins (CRISPR/Cas) (Li et al., 2014). Genome editing-based strategies for combating viral infectious diseases vary according to the characteristics of the virus and the host (Figure 1). For example, some human proteins are co-opted as viral receptors, and thus can be targets of genome editing (Li et al., 2014; Tebas et al., 2014; Xu et al., 2019). ZFNs were the first designer nucleases used to modify the *CCR5* gene, a chemokine receptor on immune cells co-opted by the HIV as a co-receptor of cellular entry, to prevent HIV infection. The CRISPR/Cas system, thanks to its ease of use and versatility, has become the dominant technology in genome editing (Anzalone et al., 2020). Various *in vitro* and animal model experiments have been performed to reduce or eliminate viral infections using the CRISPR/Cas system. Clinical studies of antiviral therapy based on CRISPR/Cas showed great promises (Tebas et al., 2014; Gupta et al., 2019; Xu et al., 2019; Anzalone et al., 2020). Here, we first offer an overview of genome editing technologies and then provide a timely update on the development of genome editing therapy for common viral infectious diseases.

## Mainstream Genome Editing Technologies

All three mainstream genome editing technologies are based on designer nucleases that exploit DNA sequence specific recognition mechanisms existing in nature. ZFNs were invented in the 1990’s and deployed as the first bona fide designer nuclease for genome editing (Kim et al., 1996; Bitinaite et al., 1998; Porteus and Carroll, 2005; Urnov et al., 2010). A ZFN consists of an array of zinc finger DNA binding motifs and a *Fok*I endonuclease domain. Each zinc finger motif recognizes three nucleotides. Two ZFNs, each containing a tandem array of 3–6 zinc finger motifs that recognize half of the target site, are required for DNA cleavage. Upon recognition of the binding sites, the *Fok*I domains of the pair of ZFNs are placed in close proximity and become activated to cleave the DNA in the middle of the two half sites (Figure 1). As the pioneering genome editing technology, ZFNs facilitated targeted introduction of desired changes in genetic materials in different organisms. However, one main concern associated with the use of ZFNs is that they may lead to off-target mutations (Porteus and Baltimore, 2003; Alwin et al., 2005; Szczepek et al., 2007). To address this problem, researchers developed several approaches, including obligate heterodimeric ZFN architectures and protein-engineering methods, to enhance the specificity of ZFNs. However, it is time-consuming to design ZFNs and empirical testing is necessary to screen for ZFNs with sufficient activity and specificity (Li et al., 2014). These constraints limit the use of ZFNs in high-throughput genome editing.

The rising interest in genome editing beckoned a rapid development of new technologies. Like the design principles of ZFNs, TALENs consist of a transcriptional activator-like effector (TALE) repeat domain and a *Fok*I nuclease domain (Figure 1). Each TALE effector repeat has two amino acids termed repeat-variable di-residues that determine its specificity for one base pair (Boch et al., 2009). As TALEs are evolved to function in tandem arrays, the modular assembly of TALENs has a much higher success rate than that of ZFNs. As such, the TALEN technology is simpler and more economical to deploy, while maintaining a high specificity, making it feasible to realize high throughput genome editing (Li et al., 2014).

The CRISPR/Cas system, whose discovery involved two decades of research by many researchers (Lander, 2016), has quickly become the most popular genome editing technology since 2012. In 1987, Ishino et al. (1987) identified the CRISPR locus when they discovered a genetic structure containing five highly homologous 29-nucleotide repeats separated by 32-nucleotide spacers. Derived from invading mobile genetics elements (MGEs), the spacer sequences are leveraged by bacteria and archaea to form an adaptive immune system (Strich and Chertow, 2019). The bacterial CRISPR system can be programmed to function in other species where a CRISPR RNA (crRNA) complementary to the target DNA and a trans-activating RNA (tracrRNA) guide the Cas nuclease to induce DNA double-strand breaks (Figure 1), followed by repair by either non-homologous end joining or homology-directed repair (Garneau et al., 2010; Deltcheva et al., 2011; Jiang and Doudna, 2017).

A diverse range of CRISPR/Cas systems with different characteristics have been described to date. For example, the CRISPR/Cas9 system targets DNA, while the CRISPR/Cas13 system targets RNA. Besides, while CRISPR/Cas9 requires the NGG protospacer adjacent motif (PAM), CRISPR/Cas13 (e.g., from *Leptotrichia wadei*) has no requirement for protospacer flanking sequences (Abudayyeh et al., 2017), which offers flexibility when targeting viruses with rapidly emerging mutations and new variants. For a detailed discussion on CRISPR/Cas genome editing tools and considerations for choosing the right tool for the application, the readers are referred to an excellent recent review (Anzalone et al., 2020).

Genome editing technologies depend on effective delivery strategies to have a desirable effect on the target biological system. Lentivirus and adeno-associated virus (AAV) are two widely utilized viral vectors for delivering genetic materials. Lentiviral vectors are easy to package and have a larger payload size than AAV vectors. An important caveat of lentiviral vectors is that they integrate into the genome of the target cell. Such permanent alterations of the host genome could potentially limit their application in basic research and in the clinic. In this regard, AAV vectors, which normally non-integrative, are generally considered to have a more desirable safety profile under normal circumstances (Athanasopoulos et al., 2017). Although recently, AAV vectors have been reported to integrate into DSBs induced by CRISPR/Cas9, raising the awareness of the risk of insertional mutagenesis when AAV is used with CRIPSR/Cas (Nault et al., 2015; Hanlon et al., 2019). Moreover, AAVs have various serotypes specific to different organs, which can be useful to target specific tissues. AAV vectors are widely used both in basic research and in clinical trials. While studies are looking into the effectiveness and safety of viral vectors, non-viral vectors are also being explored as alternatives (see Conclusion and Perspectives).

## Genome Editing Technologies as Novel Antiviral Defense

The utilization of ZFNs, TALENs, and CRISPR/Cas9 on viral infectious diseases requires the target virus to exist in a DNA form during at least part of its life cycle (Figure 1). Luckily, the major viruses that threaten human health worldwide, including human immunodeficiency virus type 1 (HIV-1), human papillomavirus (HPV), and herpes simplex virus (HSV) type 1 and 2, satisfy this requirement.

### Development of Genome Editing Therapy for HIV

HIV is a global public health burden, infecting an estimated 38 million people at the end of 2019. In HIV-1-infected patients who do not have detectable viral replication, there may still be about 10^7^ CD4^+^ T cells latently infected (Blankson et al., 2014). Because of this, although current small-molecule antiretroviral therapy (ART) may effectively inhibit HIV replication, it cannot fully eliminate the virus (Trickey et al., 2017; Kulkarni et al., 2020). Studies have shown that genome editing-based therapies can target both the active and latent HIV-1 infections (Figure 1; Hu W. et al., 2014; Yin C. et al., 2017; Yin et al., 2020).

There are different strategies to target HIV using genome editing technologies. One of them is to establish CCR5-null cells that deny HIV-1 entry (Table 1). In a pioneering study published in 2014, Tebas et al. (2014) utilized ZFN-mediated knockout of the *CCR5* gene in autologous CD4 T cells of six persons infected with HIV. The clinical trial proved the safety of ZFN-based therapy within the limits of the study, and showed the feasibility and efficiency to decrease the blood level of HIV-1 DNA (Tebas et al., 2014). In 2019, Xu et al. (2019) used the CRISPR genome editing system to ablate the *CCR5* gene *ex vivo* in CD34^+^ hematopoietic stem and progenitor cells and successfully transplanted them back to a patient with HIV and acute lymphoblastic leukemia. The patient experienced a remission of acute lymphoblastic leukemia and showed a persistence of *CCR5*-ablated donor cells for no less than 19 months (Xu et al., 2019). The overall genome editing efficiency presented in their work was modest (during the 19-month engraftment period, the frequency of *CCR5* disruption in the genome of bone marrow cells ranged between 5.20% and 8.28%). Nonetheless, genome editing endowed the edited cells with the ability to resist HIV infection, but it could not completely eliminate the virus. Nevertheless, this study is a critical step in the translation of new technologies into clinical applications, showing the feasibility and safety of genome editing therapy for HIV in clinical practice.

**TABLE 1:** Recent studies of genome editing therapy for viral infectious diseases.

**Virus**	**Context**	**Genome editing technology**	**Strategy**	**References**
HIV	Autologous CD4 T cells in people	ZFN	Lead to a five-nucleotide duplication modification (pentamer) in *CCR5*	Tebas et al., 2014
	HeLa-derived TZM-bI cells. Latently infected microglial, promonocytic, and T cells	CRISPR/Cas9	Excise a 9,709-bp fragment of integrated proviral DNA spanning from its 5′ to 3′ LTRs	Hu W. et al., 2014
	Three different animal models	CRISPR/Cas9	A quadruplex cocktail strategy to lead to multiplex fragmental deletions and multiple indel mutations in the HIV-1 provirus	Yin C. et al., 2017
	Infected human peripheral blood mononuclear cells within transgenic mouse models	CRISPR/Cas9	Remove the proviral DNA fragment from the HIV-1 viral genome within the LTRs	Bella et al., 2018
	Hematopoietic stem and progenitor cells transplanted to a patient with HIV and acute lymphoblastic leukemia	CRISPR/Cas9	Result in indels in *CCR5* that lead to *CCR5* ablation	Xu et al., 2019
	Antiretroviral therapy in non-human primates	CRISPR/Cas9	Eliminate proviral SIV DNA	Mancuso et al., 2020
	SupT1 cells	CRISPR/Cas12a	Target relatively conserved HIV sequences including LTRs	Gao et al., 2020
	HIV-1 infected HEK293T and Jurkat cells, and latently infected JLat10.6 cells	CRISPR/Cas13a	Target the conserved regions of HIV-1	Yin et al., 2020
HBV	HepG2 cells	CRISPR/Cas9	Lead to mutations and deletions in cccDNA	Seeger and Sohn, 2014
	Huh7 cells, HBV persistent mouse model	CRISPR/Cas9	Reduce the production of HBV core and surface proteins	Lin et al., 2014
	HepG2 and HeoG2.2.15 cells	CRISPR/Cas9	Target the core, polymerase, and *X* ORFs	Ramanan et al., 2015
	Huh7 cells, HeoG2.2.15 cells, mouse model carrying HBV cccDNA	CRISPR/Cas9	Target the conserved regions of HBV	Dong et al., 2015
	HepG2 and HeoG2.2.15 cells, HBV-Tg mice	CRISPR/Cas9	Target the surface antigen (HBsAg)-encoding region of HBV	Zhen et al., 2015
	Stable HBV cell line	CRISPR/Cas9	Cut a 3,175-bp HBV DNA fragment	Li et al., 2017
	Infected hNTCP-HepG2 cells	CRISPR/Cas9	Target the *S* open reading frame of HBV	Scott et al., 2017
HPV	HPV-transformed cervical carcinoma cells	CRISPR/Cas9	Target and inactivate the *E6* and *E7* oncogenes	Kennedy et al., 2014
	HPV-transformed cervical carcinoma cells	CRISPR/Cas9	Disrupt the HPV16 *E7* gene	Hu Z. et al., 2014
	HPV-transformed cervical carcinoma cells/mice	CRISPR/Cas9	Targeting promoter of HPV16 and targeting the *E6* and *E7* transcripts	Zhen et al., 2014
	HPV-transformed cervical carcinoma cells	CRISPR/Cas9	Disrupt the HPV16 *E6* gene	Yu et al., 2015
HSV-1	Vero cells	CRISPR/Cas9	Target 12 essential genes and 2 non-essential genes	van Diemen et al., 2016
	Human oligodendroglioma cells	CRISPR/Cas9	Indel mutations in exon 2 of the *ICP0* gene in the HSV-1 genome	Roehm et al., 2016
SARS-CoV-2	Synthesized fragments of SARS-CoV-2	CRISPR/Cas13d	Design and screen crRNAs targeting conserved viral regions. Identify 40 functional crRNAs targeting SARS-CoV-2	Abbott et al., 2020
	SARS-CoV-2 RNA genome data from 19 patients in China, United States, and Australia	CRISPR/Cas13d	*In silico* 10,333 guide RNAs to specifically target 10 peptide-coding regions of the *ORF1ab* and *S* genes	Nguyen et al., 2020

Another genome editing-based strategy for preventing HIV infection is to directly destroy the integrated HIV genome in latently infected cells, and to provide long-term resistance to new viral infection, expression, and replication (Table 1; Liao et al., 2015b). HIV long terminal repeats (LTRs) and the *env* and *gag* genes have been targeted to reduce and eliminate HIV in different experimental settings (Wang et al., 2018). In 2014, by bioinformatic screening and off-target prediction, Hu Z. et al. (2014) identified four CRISPR/Cas9 gRNA targets in the HIV-1 LTR promoter U3 region. By co-expressing the Cas9 protein and the four gRNAs, Hu Z. et al. (2014) efficiently inactivated HIV-1 gene expression in infected microglial and macrophage cells, the two particular cell types in the brain that harbor HIV-1. Hu Z. et al. (2014) demonstrated that their proof-of-concept work can be applicable to T cells. Liao et al. (2015a) demonstrated that engineered human induced pluripotent stem cells that stably expressed HIV-targeted CRISPR/Cas9 could differentiate into HIV reservoir cell types and maintain the resistance to HIV-1. By targeting the HIV-1 LTR promoter region, *gag*, and *pol* using sgRNA and saCas9 delivered by AAV vectors, Yin C. et al. (2017) eliminated HIV proviral DNA in various organs in three different animal models, including a humanized mouse model of chronic HIV infection. Bella et al. (2018) used a lentiviral vector to deliver a CRISPR/Cas system targeting HIV-1 LTR to infected human peripheral blood mononuclear cells to eliminate the HIV proviral DNA in humanized mouse models. By using eight male Chinese rhesus macaques with intravenous inoculation of SIV infection, a well-accepted non-human primate model of HIV infection, Mancuso et al. (2020) showed that AAV9-CRISPR/Cas9 treatment targeting the 5′ LTR-*gag* and *gag*-3′ LTR regions of the SIV genome resulted in a reduction of proviral DNA in infected blood cells and tissues. These proof-of-concept observations offer a promising step toward the elimination of HIV reservoirs in the clinic.

Besides CRISPR/Cas9, other smaller CRISPR/Cas nucleases are emerging as robust alternatives (Zetsche et al., 2015, 2017; Abudayyeh et al., 2016; Zhong et al., 2017; Paul and Montoya, 2020). Gao et al. (2020) transduced a T cell line with the CRISPR/Cas12a system using lentiviral vectors and observed a complete HIV sterilization using a single crRNA. Yin et al. (2020) utilized CRISPR/Cas13a to target the LTR, *gag*, *tat*, and *rev* regions of HIV-1 and demonstrated strong destruction of HIV-1 RNA either in invading viral capsids or expressed by latent proviruses in HEK293T, Jurkat, and JLat10.6 HIV cells. However, Cas13a recognizes and cleaves RNA, and thus cannot in theory eliminate HIV DNA and achieve a complete cure.

Due to safety concerns of genome editing, most somatic genome editing experiments were done in cells and animal models. More clinical studies of somatic genome editing are still needed.

### Development of Genome Editing Therapy for HBV, HPV, and HSV

More than 250 million people are living with chronic hepatitis B virus (HBV) infection (Strich and Chertow, 2019). Though effective prophylactic HBV vaccines are available, due to the highly stable HBV covalently closed circular DNA (cccDNA) existing in the nuclei of infected cells, effective therapies to eliminate the virus remain elusive. Recent studies showed that CRISPR/Cas could be a potential effective treatment for HBV infection (Table 1). Scott et al. (2017) successfully inactivated cccDNA in HBV-infected hNTCP-HepG2 cells by using the CRISPR/Cas system to target the *S* open reading frame (ORF) of HBV. In the same year, Li et al. (2017) utilized CRISPR/Cas9 to cut a 2,175-bp HBV DNA that integrated into the genome of infected cells. In these experiments, AAV vectors, including AAV8 and its derivatives, showed a good tropism for liver, making them a promising vector for delivering the CRISPR/Cas anti-HBV therapy.

More than 150 different HPV serotypes can infect humans. Though most of them are harmless or only induce benign warts, a small group of the serotypes are regarded as high-risk factors for cancer. Epidemiological studies show that HPV16 and HPV18 account for about 75% of cervical cancer, while HPV31 and HPV45 account for a further 10%. Besides, HPVs are significantly associated with oropharyngeal and anal cancers. When HPVs overexpress the E6 and E7 proteins that inhibit cellular tumor suppressors, the risk of malignant transformation for the infected cells increases (Werness et al., 1990; Hu Z. et al., 2014; Zhen et al., 2014). It has shown that loss of E6 and E7 function induces p53 and Rb expression, respectively, and thus, induces cell cycle arrest. Inspired by this, several groups explored the possibility of utilizing CRISPR/Cas-mediated cleavage to disturb HPV *E6* or *E7* genes (Table 1). Kennedy et al. (2014) used CRISPR/Cas9-based strategy to target the amino-terminal regions of HPV18 *E6* and *E7* ORFs and successfully inactivated the *E6* and *E7* genes in cervical carcinoma cells transformed by HPV18. Hu Z. et al. (2014) demonstrated that targeting the *E7* gene of HPV16 with CRISPR/Cas could destroy *E7* DNA and induce apoptosis and growth inhibition of HPV-positive SiHa and Caski cells while sparing HPV-negative C33A and HEK293 cells. Besides, the disruption of *E7* DNA lead not only to down-regulated E7 protein but also to up-regulated pRb, a tumor suppressor protein (Hu Z. et al., 2014). Zhen et al. (2014) transduced the CRISPR/Cas9 system targeting the promoter and transcripts of HPV16 *E6/E7* into HPV16-positive SiHa cells and observed increased levels of p53 and p21 proteins. These efforts showed genome editing as an effective path to eliminate HPV DNA, down-regulate the expression of the two oncogenes *E6* and *E7*, and up-regulate tumor suppressors *p53* and *p21*, suggesting the promise of CRISPR/Cas for treating HPV infectious diseases (Hu Z. et al., 2014; Kennedy et al., 2014; Zhen et al., 2014; Yu et al., 2015).

HSV is another virus that spreads around the world. van Diemen et al. (2016) reduced HSV-1 replication in Vero cells by targeting 14 different genes using the CRISPR/Cas9 system. Targeting multiple genes using genome editing shows a greater efficiency of HSV-1 elimination than targeting single genes (van Diemen et al., 2016). Roehm et al. (2016) suppressed HSV-1 replication in human oligodendroglioma cells by inducing mutations into the *ICP0*, *ICP4*, and *ICP27* genes that are important for viral replication.

In addition to the above-mentioned cases, proof-of-concept laboratory studies have also shown that genome editing technologies can target and destroy other viruses including lymphocytic choriomeningitis virus, influenza A virus, and vesicular stomatitis virus (Freije et al., 2019). Such versatility of genome editing-based antiviral treatment is perhaps best exemplified by the rapid research progress toward the prevention of SARS-CoV-2 infection using CRISPR, which deserves a separate discussion below.

### Development of Genome Editing Therapy for SARS-CoV-2

SARS-CoV-2 has been rapidly spreading around the world and has caused more than 2.5 million deaths at the time of writing. While several vaccines have shown good efficacies in preventing COVID-19, vaccine supply shortages, emerging mutant variants around the world, inadequate immune response in immunocompromised individuals, and rare side effects still leave hundreds of millions of people exposed to the risk of contracting SARS-CoV-2. Available small molecule antiviral drugs have limited effects on SARS-CoV-2. Although monoclonal antibodies are cleared by the US FDA for emergency use, they are quite expensive and are only authorized for mild to moderate COVID-19 cases and may be associated with worse outcomes when administered to severe cases that require hospitalization. Thus, new anti-viral treatments for SARS-CoV-2 are urgently needed.

Recently, the CRISPR/Cas13 system has been under the spotlight since it targets RNA and shows a great potential in treating RNA viral infectious diseases, including COVID-19. Researchers have used CRISPR/Cas13-based technology to develop novel antiviral drugs to combat SARS-CoV-2 (Table 1; Abbott et al., 2020; Nguyen et al., 2020). By targeting highly conserved regions of the SARS-CoV-2 genome using a gRNA, Cas13 can cut and clear the viral RNA genome. The action of the CRISPR system can be organ specific by leveraging AAV serotypes that are specific to the lung. Besides, researchers can apply multiple gRNAs to ensure successful targeting of the virus even if part of it mutates. To help develop a CRISPR/Cas13d system that specifically eliminates SARS-CoV-2 genome, Nguyen et al. (2020) designed 10,333 gRNAs to target ten coding regions of the *ORF1ab* and *S* genes in the SARS-CoV-2 genome and proposed AAV to be the vector to simultaneously deliver up to three gRNAs targeting different coding regions of SARS-CoV-2. The authors claimed that the gRNAs did not affect the human transcriptome, though experimental data should be added to support the efficacy and safety of their system (Nguyen et al., 2020). Abbott et al. (2020) established a prophylactic antiviral CRISPR in human cells (PAC-MAN) strategy that uses CRISPR/Cas13d to degrade viral sequences. The PAC-MAN strategy proved to be capable of inhibiting SARS-CoV-2 fragment expression as well as influenza A virus infection in human lung epithelial cells (Abbott et al., 2020). Additionally, they utilized bioinformatics analysis to predict groups of CRISPR-associated RNAs to target all sequenced coronaviruses, though the effectiveness and *in vivo* safety of this strategy need to be examined before putting it into clinical use (Abbott et al., 2020). To expand their work, Lin et al. (2021) applied the PAC-MAN strategy to a broad spectrum of human- or livestock-tropic RNA viruses. The *in silico* analysis showed that a minimal set of 14 crRNAs can target >90% of human-tropic viral genomes. Furthermore, by co-transfecting plasmids containing one of five predicted crRNAs and SARS-CoV-2 reporters into A549 lung epithelial cells, Lin et al. (2021) validated the PAC-MAN strategy. Specifically, they observed that the decrease in the percentage of SARS-Cov-2 reporter-positive cells correlated significantly with the predicted targeting efficiency score of crRNAs and with the viral RNA abundance. These results validated their prediction algorithm for the targeting efficiency of crRNAs for the viral genome (Lin et al., 2021).

While individual crRNA showed an ability to target almost 99% of SARS-CoV-2 isolates, the pooling of the five experimentally validated crRNAs could target all of the SARS-CoV-2 variants as identified in GISAID on February 3, 2021 (Lin et al., 2021). Moreover, Lin et al. (2021) have rolled out an online resource^1^ for the use of CRISPR/Cas13 to target RNA viruses, which when combined with rapid field-deployable virus sequencing (Bi et al., 2021) could greatly speed up the design of genome editing tools for combating emerging viruses. These proof-of-principle studies highlight the versatility and flexibility of CRISPR as an intracellular defense against many types of RNA viruses, including but not limited to SARS-CoV-2.

## Conclusion and Perspectives

The flexible characteristic of genome editing technologies, especially the CRISPR/Cas system, gives it an inherent advantage when dealing with the fast evolution of mutations in viral pathogens. Though clinically approved genome editing therapies are not yet available, early clinical trials are ongoing. Safety problems, including immunogenicity and mutagenesis, still need investigation. Recent reports showed that DNA repair following the double strand breaks induced by the CRISPR/Cas system can cause unintended mutations including large structural variations (Kosicki et al., 2018; Bi et al., 2020). Furthermore, Haapaniemi et al. (2018) showed that CRISPR/Cas9 can induce p53-mediated DNA damage response and leads to a selection against p53 proficient cells. Since p53-mediated mechanisms are key to maintaining genome stability, potential tumorigenesis originated from genome-edited cells remains a concern and needs to be further examined.

Efficient and safe delivery of genome editing technologies remains a challenge, especially for *in vivo* applications. To this end, Yin H. et al. (2017) developed an optimal set of chemical modifications in sgRNAs to maintain or enhance genome-editing efficiency for *in vivo* applications. A single intravenous injection of the enhanced sgRNAs with CRISPR/Cas9-based lipid nanoparticle formulations into mice induced >80% disruption of *Pcsk9* in the liver and achieved undetectable levels of Pcsk9 in the serum (Yin H. et al., 2017). Alsaiari et al. (2018) developed nanoscale zeolitic imidazole frameworks (ZIFs) to deliver CRISPR/Cas9 to mammalian cells as nanoparticles and showed CRISPR/Cas-ZIFs are biocompatible and offer efficient co-delivery of Cas9 protein and sgRNA. Mangeot et al. (2019) developed a vehicle called nanoblades by loading Cas9-sgRNA ribonucleoproteins onto engineered murine leukemia virus-like particles. Nanoblades targeting *Hpd* achieved between 7% and 13% targeting efficiency in mouse liver 2 weeks after retro-orbital injection and showed no signs of morbidity (Mangeot et al., 2019). The choice of vector is still under discussion. As mentioned before, AAV2 showed insertional mutagenesis in human hepatocellular carcinomas (Nault et al., 2015). Besides, the current evidence clearly demonstrates that AAV vectors can trigger innate and adaptive immune responses, leading to transgene loss (Mingozzi and High, 2013; Ronzitti et al., 2020; Samelson-Jones et al., 2020). Vector design and the total dose of AAVs need to be optimized to decrease the immune-mediated toxicity before clinical use (Ling Li et al., 2015; Ronzitti et al., 2020).

Ethical concern of human genome editing is a hotly debated issue (Isasi et al., 2016). Genome editing therapies for viral pathogens carry the risk of altering the human genome. The current consensus recommendation is that genome editing in somatic cells is acceptable if the goal is to treat severe diseases, while germline editing is strongly discouraged (National Academies of Sciences, 2017; National Academy of Sciences, 2020). In this sense, genome editing therapies for viral infectious diseases are likely to encounter less ethical issues as they only target somatic cells.

In sum, exciting advances have been made in the development of genome editing as treatment for viral infectious diseases, but significant challenges lie before clinical translation of the research. Some of these challenges, such as safety and efficiency of CRISPR, are common to genome editing technologies and are being addressed by the genome editing field, while others require a commitment to progress from proof-of-concept studies to preclinical animal models and ultimately to clinical trials. With the COVID-19 pandemic raging through the world and causing human tragedies and economic devastations globally, now may just be the right time to commit to make genome editing therapy for infectious disease a clinical reality.

## Author Contributions

YZ reviewed the literature and drafted the manuscript. ML revised the manuscript and supervised the study. YZ and ML conceived the study. Both authors contributed to the article and approved the submitted version.

## Conflict of Interest

The authors declare that the research was conducted in the absence of any commercial or financial relationships that could be construed as a potential conflict of interest.

## Footnotes

1 crispr-pacman.stanford.edu
